# Herbal medicine as a complementary therapy for dysmenorrhea: effects on pain and reduction of analgesic use

**DOI:** 10.3389/fmed.2026.1719823

**Published:** 2026-05-15

**Authors:** Soo-Hyun Sung, Kyeore Bae, Hyein Jeong, Dong-Il Kim, Minjung Park

**Affiliations:** 1 School of Korean Medicine, Wonkwang University, Iksan, Republic of Korea; 2 National Agency for Korean Medicine Innovative Technologies Development, National Institute for Korean Medicine Development, Seoul, Republic of Korea; 3 Department of Preventive Medicine, College of Korean Medicine, Kyung Hee University, Seoul, Republic of Korea; 4 Department of Obstetrics and Gynecology, Dongguk University Ilsan Korean Medicine Hospital, Goyang, Republic of Korea; 5 Department of Preventive Medicine, College of Korean Medicine, Gachon University, Seongnam, Republic of Korea

**Keywords:** dysmenorrhea, herbal decoction, herbal medicine, real-world evidence, registry study

## Abstract

**Objective:**

Dysmenorrhea is a prevalent gynecological disorder impairing quality of life for women of reproductive age. Although traditional herbal decoctions (THDs) are widely used for dysmenorrhea in Korea, robust real-world evidence regarding their clinical outcomes and safety is limited. This descriptive, hypothesis-generating registry study aimed to examine individualized THDs for dysmenorrhea using registry data from a multicenter observational study.

**Methods:**

This multicenter, prospective observational registry study enrolled women aged 19–65 years with dysmenorrhea from 33 traditional Korean medicine (TKM) clinics and one TKM hospital in Korea (July 2022–June 2023). Participants were categorized into a THD group (received THDs for ≥10 days) and a non-THD group. Primary outcomes were changes in pain intensity (Numeric Rating Scale, NRS), duration of menstrual pain, and analgesic consumption (defined as the number of analgesic tablets taken per menstrual cycle), assessed at each visit. To address confounding and selection bias, a difference-in-differences analysis using inverse probability of treatment weighting (IPTW) based on propensity scores was performed. Missing data were handled using complete-case analysis, supported by Little’s MCAR test (*p* = 0.39). Safety was assessed by monitoring and categorizing adverse events.

**Results:**

A total of 119 participants in the THD group and 16 in the non-THD group completed the study. Both groups demonstrated significant within-group reductions in menstrual pain intensity over time. Regarding between-group differences, the THD group showed a significantly greater observed reduction in menstrual pain intensity at visit 2 compared to the non-THD group (Estimate = −1.41, *p* = 0.029); no significant between-group difference was observed at other time points. Analgesic use also showed a statistically significant observed decrease in the THD group at visit 2 (Estimate = −1.63, *p* = 0.001). No statistically significant between-group difference was identified for pain duration at any time point. Mild adverse events were reported by 9 participants (7.6%) in the THD group; all resolved spontaneously. No adverse events occurred in the non-THD group.

**Conclusion:**

These preliminary findings suggest that individualized THDs may be associated with reduced menstrual pain intensity and analgesic consumption in women with dysmenorrhea in real-world settings; however, significant between-group differences were observed at a single time point only, pain duration did not differ significantly between groups, and comparative safety conclusions remain constrained by the small control group. Larger, adequately powered studies are needed to confirm these exploratory findings.

## 1 Introduction

Despite the high prevalence rate among reproductive women, dysmenorrhea is an undertreated gynecological disorder (1–3). Dysmenorrhea is clinically characterized by recurrent crampy pelvic pain associated with the menstrual cycle, which may be variably accompanied by nausea, diarrhea, fatigue, irritability, and nervousness (4, 5). Unmanaged dysmenorrhea impairs daily activities and leads to school and work absenteeism (6, 7). Given that 75% of the cost of chronic pelvic pain without endometriosis is attributed to productivity loss, dysmenorrhea may pose a substantial indirect economic burden exceeding direct healthcare costs (8). Moreover, the prevalence of dysmenorrhea has increased from 58.8% [95% confidence interval (CI): 54.3–63.7] before 2010 to 71.5% (95% CI: 66.8–76.6%) during 2015–2021 (9), potentially attributable to multifactorial contributors including lifestyle changes, psychological factors, and underlying organic pathologies such as endometriosis, uterine fibroids, and pelvic inflammatory disease, which may interact synergistically to intensify symptom severity (10–13). This upward trend highlights the growing need for effective management of dysmenorrhea.

The primary goal of dysmenorrhea management is symptom relief. As first-line treatments, NSAIDs and hormonal contraceptives are used empirically for up to three months; topical heat therapy and exercise are additionally recommended as baseline interventions. If the symptoms persist, further evaluation—including imaging studies or laparoscopy—is required to identify underlying abnormalities (4, 5, 14). The 2025 clinical practice guideline (CPG) by the Society of Obstetricians and Gynaecologists of Canada recommends acupuncture, acupressure, and oral ginger supplementation as complementary treatments for primary dysmenorrhea (14). In Japan, the most commonly prescribed first-line treatments were herbal medicine extracts (38.8%) and low-dose estrogen progestins (LEPs, 27.4%), and LEPs (50.2%) and herbal medicine extracts (19.5%) for secondary dysmenorrhea (15). In the Republic of Korea, herbal medicine is widely used within traditional Korean medicine (TKM) practice for dysmenorrhea management, as recommended by CPGs based on randomized controlled trials (RCTs) (16).

Since the implementation of the THD National Health Insurance (NHI) Benefit Coverage Extension Pilot Project in November 2020, individualized THDs for dysmenorrhea have been covered under NHI reimbursement in South Korea (17). The customized nature of these THDs poses challenges in evaluating their safety and efficacy through RCTs. A recent nationwide retrospective study using NHI claims data reported a significant reduction in dysmenorrhea severity over time following THD treatment (18). However, due to the lack of key covariates such as obesity, smoking status, and parity in claims data, the study could not adequately adjust for potential effect modifiers, limiting the conclusiveness of the findings (5, 19).

To address these limitations and examine potential associations between THD use and clinical outcomes, we conducted a multicenter prospective registry study on THDs for dysmenorrhea employing difference-in-differences analysis with inverse probability of treatment weighting (IPTW) as an analytical framework to explore potential associations. This study is primarily descriptive and hypothesis-generating in nature; it aimed to characterize the potential associations between individualized THD use and outcomes related to pain and analgesic consumption within the NHI pilot project framework, and to identify research questions warranting investigation in future adequately powered confirmatory trials.

## 2 Methods

### 2.1 Study design/setting

This was a descriptive, hypothesis-generating, prospective observational multicenter study of patients receiving TKM treatment for dysmenorrhea in South Korea. The study was conducted at a total of 34 sites, including 33 Korean medicine clinics and 1 Korean medicine hospital nationwide. Participants were female patients who visited these institutions for dysmenorrhea management. At baseline of enrollment, comprehensive data were collected on a range of variables, including demographic characteristics, anthropometric measurements, health behavior factors, prescription details, concurrent treatments, NRS scores, duration of menstrual pain, and analgesic use. An electronic Case Report Form (eCRF) was developed using the public eCRF system for Korean Medicine research, provided by the National Agency for Korean Medicine Innovative Technology Development (KoMIT) (20).

### 2.2 Eligibility criteria

#### 2.2.1 Inclusion criteria

1. Women aged between 19 and 65 years old.
2. Individuals who visited Korean medicine clinics or the hospital for the treatment of dysmenorrhea and elected to receive traditional Korean medicine treatment.
  - The group receiving THD treatment was designated as the experimental group, while the group not receiving THD treatment served as the control group (non-THD).
  - The use of other Korean medicine therapies related to dysmenorrhea (e.g., acupuncture, moxibustion, cupping) and conventional medicine treatments is permitted.
3. Patients who received a detailed explanation prior to enrollment, voluntarily agreed to participate in the study, and provided written informed consent approved by the Institutional Review Board of Dongguk University (DUIOH 2022–07–008-002).

#### 2.2.2 Exclusion criteria

1. Individuals who do not agree to participate in the registry.
2. Patients receiving treatment for conditions other than dysmenorrhea (e.g., secondary dysmenorrhea due to pelvic inflammatory disease, endometriosis, uterine fibroids, or other identifiable pelvic pathologies).
3. Individuals with chronic diseases that may affect the interpretation of treatment outcomes (e.g., cardiovascular disease, dementia, psychiatric disorders), pregnant women, or those deemed unsuitable for participation by the investigator, or those with abdominal conditions that may interfere with treatment or assessment, including recent abdominal wounds (within 1 month), active abdominal infections, or severe skin diseases at the treatment sites.
4. Individuals who have used THDs or compound preparations containing similar ingredients to THDs within the past 1 month.

### 2.3 Recruitment

Participants were recruited from 33 Korean medicine clinics and 1 Korean medicine hospital nationwide from July 2022 to June 2023. Prior to enrollment, researchers provided a detailed explanation of the study objectives, data usage, and confidentiality procedures. Written informed consent was obtained from all eligible participants; for participants under 18 years of age, consent was provided by a parent or legal guardian. Recruitment posters were displayed at the entrances and inside the elevators of each clinic and hospital to facilitate participants’ enrollment.

A total of 135 participants were enrolled in the study. Among them, 119 patients who had taken herbal medicine for more than 10 days were assigned to the intervention group, and 16 patients who did not take herbal medicine were assigned to the control group. The allocation was based on the participants’ actual treatment choices during routine clinical practice.

### 2.4 Interventions

Participants in the THD group received individualized THD treatment. THD was administered orally twice per day for a duration of at least 10 days. The selection of herbal prescriptions was guided by the Korean Medicine CPG for Dysmenorrhea (16). This evidence-based CPG, developed using systematic literature review, expert consensus from domestic and international panels, and Grading of Recommendations Assessment, Development and Evaluation (GRADE) methodology, provides diagnostic criteria and treatment recommendations for dysmenorrhea. All herbal ingredients were prepared in accordance with the Good Manufacturing Practice (GMP) standards for herbal medicinal products.

### 2.5 Variables

Data were collected on demographics, health behavior factors, prescription information, concurrent treatments, effectiveness outcomes, and safety outcomes to evaluate the impact of THD treatment for dysmenorrhea (Table 1). All categories were collected for the THD group; for the non-THD group, THD-related categories were excluded. Data were collected at enrollment (Visit 0) and at the end of each subsequent menstrual cycle (Visit 1, Visit 2, and Visit 3). Analgesic consumption was defined as the total number of analgesic tablets or capsules taken per menstrual cycle, as self-reported by participants at each visit, regardless of analgesic type or dose.

**Table 1:** Data collected from participants.

Category	Detailed information
Demographics	Age, Height, Weight, Body mass index
Health Behavior Factors	Marital status, Smoking status, Number of Term Births, Number of Preterm Births, Number of Abortions, Number of Living Children, Past history, Category of dysmenorrhea
Prescription Information of THD	Composition of Herbal Decoction
Concurrent Treatments	Use of acupuncture, use of Analgesics
Effectiveness Outcome Measures	NRS, Duration of Menstrual Pain, Analgesic Consumption
Safety Outcome Measures	Presence or Absence of Adverse Events, Detailed Symptoms of Adverse Events

NRS, Numeric Rating Scale; THD, Traditional herbal decoction.

### 2.6 Data management and quality control

Data collection was conducted using the eCRF system. Licensed Korean medicine doctors at each participating clinic reviewed the clinical assessment results for each participant at every visit and subsequently entered the data into the eCRF system. To access the data entry platform, clinicians connected to the web server^1^ via an internet-enabled device, where they logged in using credentials provided by the system administrator. Each registry entry underwent periodic monitoring and quality checks, with data queries issued by clinical research associates in accordance with the study’s data monitoring plan to ensure accuracy. All study data were securely stored on a dedicated server with restricted access privileges to protect participants’ confidentiality.

### 2.7 Safety assessment

Safety outcomes were evaluated by documenting adverse events reported in both the THD and non-THD groups throughout the study period. All reported events were graded in accordance with the Common Terminology Criteria for Adverse Events (CTCAE) (21). The severity levels were defined as follows: Grade 1 (mild), characterized by asymptomatic or mild clinical findings not requiring therapeutic intervention; Grade 2 (moderate), involving minimal or noninvasive management and potential limitations in instrumental activities of daily living appropriate to age; Grade 3 (severe), referring to medically significant events that were not immediately life-threatening but required hospitalization or extended hospital stay and restricted self-care activities of daily living; Grade 4 (life-threatening), indicating conditions necessitating urgent medical intervention; and Grade 5, defined as death attributable to the adverse event.

### 2.8 Statistical analyses

To compare baseline characteristics between THD group and non-THD group, student’s t- test or Wilcoxon rank sum test for continuous data, and Chi-squared test or Fisher’s Exact test for categorical data were conducted. To address selection bias arising from the non-randomized study design, a difference-in-differences (DID) analysis using IPTW based on propensity scores was conducted (22). Propensity scores were estimated using logistic regression based on multiple covariates, including demographic factors, gynaecological history, baseline severity, and concurrent acupuncture treatment. IPTW was employed to adjust heterogeneity between THD and non-THD groups (23), controlling for time-invariant observed confounders. To explore potential associations between THD use and pain severity, duration, and analgesic dose, we performed a weighted linear regression analysis using time (visit), treatment group, and the interaction term between time and treatment group as key variables, where the coefficient of the interaction term represented the DID effect. Residual imbalances following IPTW were addressed by including corresponding variables as covariates in the DID analyses. Safety was assessed by the number and rate of adverse events reported. Analyses were conducted using available data at each time point, without imputation for missing values, since missing at completely random was not denied through Little’s MCAR test (*p* = 0.39). Statistical significance was set at *p <* 0.05. Statistical analyses were conducted using STATA MP Ver. 14.

### 2.9 Ethics and dissemination

This study was approved by the institutional review Board of Ilsan Korean Medicine Hospital, Dongguk University (DUIOH 2022–07–008-002). All participants provided written informed consent prior to enrollment.

## 3 Results

### 3.1 Participant characteristics

At baseline, no statistically significant differences were observed between the two groups, except for past medical history and dysmenorrhea type, both of which may influence treatment outcomes and preclude direct group comparison without further adjustment.

To improve comparability between groups, propensity scores were calculated using covariates associated with both THD use and potential menstrual pain outcomes.

No significant differences were observed between groups in demographic or reproductive characteristics. However, several clinical variables differed significantly: the THD group had a significantly higher proportion of participants with a history of medical conditions (*p* = 0.04). Furthermore, the THD group showed a higher analgesic consumption with borderline statistical significance (*p* = 0.06). The THD group had a significantly longer duration of menstrual pain (*p <* 0.01), and greater average pain intensity (NRS, *p <* 0.01) compared to the control group, suggesting that participants in the THD group presented with more severe menstrual symptoms at baseline (Table 2).

**Table 2:** Baseline characteristics of patients before and after inverse probability of treatment weighting (IPTW) adjustment.

Patient characteristics	Before IPTW				After IPTW			
	THD	Non-THD	*p*-value	SMD	THD	Non-THD	*p*-value	SMD
Mean age, year (SD)	28.23 (11.38)	34.06 (15.54)	0.07	0.39	28.77 (11.63)	27.42 (12.26)	0.69	0.10
Marital Status, *n* (%)			<0.01	0.37			0.01	0.13
Yes	38 (31.93)	8 (50.00)			22.45 (34.51)	17.11 (25.56)		
No	81 (68.07)	8 (50.00)			42.60 (65.49)	49.84 (74.44)		
Mean body mass index, kg/m^2^ (SD)	20.90 (3.27)	21.79 (3.78)	0.31	0.22	20.98 (3.28)	21.25 (3.35)	0.77	0.05
Alcohol intake, *n* (%)			1.00	0.19			0.69	0.19
Yes	40 (33.6)	10 (62.5)			46.4 (35.2)	56.5 (41.7)		
No	79 (66.4)	6 (37.5)			85.3 (64.8)	79.05 (58.3)		
Number of term births, *n* (%)			0.06	0.40			0.23	0.17
0	89 (74.8)	9 (56.2)			95.5 (72.5)	111.9 (82.6)		
1	13 (10.9)	1 (6.2)			14.7 (11.2)	3.4 (2.54)		
2	13 (10.9)	4 (25.0)			16.9 (12.9)	12.5 (9.2)		
3	2 (1.7)	2 (12.5)			2.3 (1.7)	7.7 (5.7)		
4	2 (1.7)	0 (0.0)			2.2 (1.7)	0 (0.0)		
Number of preterm births, *n* (%)			0.81	0.21			0.23	0.21
0	116 (97.5)	16 (100.0)			128.4 (97.5)	135.55 (100.0)		
1	2 (1.7)	0 (0.0)			2.2 (1.6)	0 (0.0)		
2	1 (0.8)	0 (0.0)			1.2 (0.9)	0 (0.0)		
Use of acupuncture, *n* (%)			0.32	0.37			0.96	0.47
Yes	24 (20.2)	1 (6.2)			25.9 (19.6)	25.5 (18.8)		
No	95 (79.8)	15 (93.8)			105.9 (80.4)	110.05 (81.2)		
Mean analgesic use, *n* (SD)	3.17 (3.64)	1.38 (1.75)	0.06	0.34	2.90 (3.53)	2.35 (2.12)	0.50	0.04
Mean average pain intensity, NRS (SD)	6.24 (1.98)	4.56 (2.10)	<0.01	0.84	6.06 (2.05)	6.23 (2.07)	0.80	0.01

IPTW, Inverse probability of treatment weighting; NRS, Numeric rating scale; SD, Standard deviation; THD, Traditional herbal decoction; SMD, Standardized Mean Difference. Values for SMD are presented as absolute values.

Following IPTW, the THD group and non-THD group were well-balanced in most characteristics (SMD < 0.2). However, number of preterm birth and acupuncture treatment remained unbalanced. These variables were further adjusted for in the DID analysis to ensure comparability across groups.

### 3.2 Traditional herbal medicine

A total of 26 types of THD prescriptions were used among the 119 participants in the THD group. Among these, three prescriptions—Hyeonburikyung-tang-gagam, Gyejibokryeong-hwan-gami, and Sobokchukeo-tang—were administered to more than 10 participants (Table 3).

**Table 3:** Frequently used herbal prescriptions in the experimental group.

Names of herbal decoction	Composition and dosage of herbal medicines	*N*	%
Hyeonburikyung-tang-gagam*	*Cyperi Rhizoma* 4.0 g*, Corydalis Tuber* 4.0 g*, Atractylodis Rhizoma Distichae* 4.0 g*, Linderae Radix* 4.0 g*, Citri Unshiu Pericarpium* 4.0 g*, Angelicae Gigantis Radix* 4.0 g*, Paeoniae Radix Alba* 4.0 g*, Cnidii Rhizoma* 4.0 g*, Aurantii Fructus Immaturus* 3.0 g*, Curcumae Rhizoma* 3.0 g*, Persicae Semen* 3.0 g*, Cinnamomi Cortex* 3.0 g*, Aucklandiae Radix* 3.0 g*, Carthami Flos* 2.0 g*, Zingiberis Rhizoma Recens* 3.0 g	52	43.70
Gyejibokryeog-hwan-gami*	*Cinnamomi Ramulus* 4.0 g*, Paeoniae Radix* 4.0 g*, Moutan Cortex* 4.0 g*, Persicae Semen* 4.0 g*, and Poria* 4.0 g	21	17.65
Sobokchugeo-tang	*Foeniculi Fructus* 4.0 g*, Zingiberis Rhizoma Recens* 4.0 g*, Corydalis Tuber* 4.0 g*, Angelicae Sinensis Radix* 4.0 g*, Chuanxiong Rhizoma* 4.0 g*, Paeoniae Radix* 4.0 g*, Typhae Pollen* 3.0 g*, Trogopterorum Faeces* 3.0 g*, Cinnamomi Cortex* 3.0 g*, and Myrrha* 3.0 g	11	9.24

*The term ‘Gagam/Gami’ refers to the clinical practice in Korean Medicine where a Korean medicine doctor adjusts the composition and dosage of herbs in a classical formula based on the patient’s specific symptoms and constitution.

### 3.3 Effectiveness outcomes

Tables 4–6 present the estimated effects of THD on three menstrual pain–related outcomes (pain severity, pain duration, and analgesic consumption) over time. To account for repeated measures nested within participants, clustered robust standard errors were applied. Covariates including age, marital status, smoking, birth history, dysmenorrhea classification, baseline pain (NRS), baseline medication use, and acupuncture treatment were incorporated to adjust for baseline disparities. Full regression results are provided in Tables 4–6.

**Table 4:** Estimated effects of herbal medicine on severity of menstrual pain (NRS).

	Variation	Estimate	Std. error	*t* value	*p*-value	CI lower	CI upper
Severity of menstrual pain (NRS)	Age	0.00	0.01	0.25	0.80	−0.02	0.03
	Marriage	−0.04	0.47	−0.08	0.94	−0.96	0.89
	Smoking	−0.65	0.68	−0.95	0.34	−1.99	0.70
	Full-term birth	−0.53	0.49	−1.08	0.28	−1.50	0.44
	Preterm birth	0.82	0.25	3.30	<0.01*	0.33	1.31
	Primary/Secondary	0.37	0.46	0.81	0.42	−0.54	1.29
	NRS at baseline	0.66	0.08	8.65	<0.01*	0.51	0.81
	Analgesic use at baseline	0.02	0.03	0.49	0.63	−0.05	0.08
	Acupuncture Tx.	−0.27	0.26	−1.02	0.31	−0.78	0.25
	Visit						
	Visit 1	−1.35	0.36	−3.77	<0.01*	−2.06	−0.64
	Visit 2	−1.65	0.52	−3.15	<0.01*	−2.69	−0.61
	Visit 3	−3.08	0.80	−3.87	<0.01*	−4.66	−1.51
	Group (ref. =non-THD)						
	THD group	−0.17	0.24	−0.72	0.47	−0.63	0.30
	Interaction effect						
	Herbal: Visit 1	−0.75	0.43	−1.75	0.08	−1.59	0.10
	Herbal: Visit 2	−1.41	0.64	−2.21	0.03*	−2.68	−0.15
	(Intercept)	3.18	2.01	1.58	0.12	−0.80	7.16
Linear regression Number of obs = 338 *F*(15, 131) = 23.10 Prob > *F* = 0.0000 R-squared = 0.5971 Root MSE = 1.6215 (Std. Err. adjusted for 132 clusters in a_newID)							

*p < 0.05.

To minimize the influence of extreme weights and enhance the stability of our estimates, we conducted a sensitivity analysis by trimming weights at the 99th percentile. Weights exceeding this threshold were winsorized to the 99th percentile value. The results were consistent with the original estimates, and the full comparative data are provided in Supplementary material.

#### 3.3.1 Severity of menstrual pain

A total of 338 observations from 132 unique participants were analyzed. Standard errors were adjusted for 132 clusters to provide valid inference despite the intra-cluster correlation inherent in repeated measures. Menstrual pain severity (NRS) decreased significantly over time. Compared to baseline (visit 0), significant reductions were observed at visit 1 (*Estimate* = −1.35, *p* < 0.01), visit 2 (*Estimate* = −1.65, *p* < 0.01), and visit 3 (*Estimate* = −3.08, *p* < 0.01). The main effect of group was not statistically significant, indicating no overall difference in baseline pain severity between the THD and control groups (*Estimate* = −0.17, *p* = 0.47). The DID interaction analysis showed a significantly greater reduction in pain severity in the THD group at visit 2 compared to the control group (*Estimate* = −1.41, *p* = 0.03), whereas the difference at visit 1 was not statistically significant (*Estimate* = −0.75, *p* = 0.08) (Table 4).

#### 3.3.2 Duration of menstrual pain

The final regression model included 355 observations from 132 participants. We utilized a cluster-robust estimator to account for the lack of independence between repeated measurements within the same individual. Pain duration decreased significantly over time. Compared to baseline (visit 0), significant reductions were observed at visit 1 (*Estimate* = −13.81, *p* = 0.02), visit 2 (*Estimate* = −13.68, *p <* 0.01), and visit 3 (*Estimate* = −14.62, *p* = 0.01). Between-group comparison revealed that the herbal medicine group had a significantly longer duration of menstrual pain than the control group (*Estimate* = 9.40, *p <* 0.05). However, no statistically significant interaction effects were observed at visit 1 or visit 2 (*p* > 0.7), indicating no significant between-group difference in pain duration (Table 5).

**Table 5:** Estimated effects of herbal medicine on duration of menstrual pain.

	Variation	Estimate	Std. error	*t* value	*p*-value	CI lower	CI upper
Duration of menstrual pain	Age	−0.13	0.13	−0.95	0.34	−0.39	0.14
	Marriage	2.17	3.81	0.57	0.57	−5.37	9.71
	Smoking	7.58	3.76	2.02	0.05*	0.14	15.01
	Full-term birth	−0.30	4.30	−0.07	0.94	−8.82	8.21
	Preterm birth	3.65	1.63	2.24	0.03*	0.42	6.87
	Primary/Secondary	−0.18	3.94	−0.05	0.96	−7.97	7.60
	NRS at baseline	−0.25	0.62	−0.40	0.69	−1.47	0.98
	Analgesic use at baseline	2.52	0.58	4.38	<0.01*	1.38	3.66
	Acupuncture Tx.	4.90	3.51	1.39	0.17	−2.05	11.85
	Visit						
	Visit 1	−13.808	5.922	−2.33	0.02*	−25.524	−2.092
	Visit 2	−13.679	4.156	−3.29	<0.01*	−21.901	−5.458
	Visit 3	−14.622	5.065	−2.89	0.01*	−24.642	−4.603
	Group (ref. =non-THD)						
	THD group	9.403	4.690	2.01	0.05*	0.126	18.681
	Interaction effect						
	Herbal: Visit 1	−2.363	6.390	−0.37	0.71	−15.005	10.279
	Herbal: Visit 2	−1.378	5.439	−0.25	0.80	−12.139	9.382
	(Intercept)	−3.942	12.909	−0.31	0.76	−29.479	21.596
Linear regression Number of obs = 355 *F*(15, 131) = 11.35 Prob > F = 0.0000 R-squared = 0.3148 Root MSE = 17.948 (Std. Err. adjusted for 132 clusters in a_newID)							

*p < 0.05.

#### 3.3.3 Analgesic consumption

The final regression model included 336 observations from 132 participants. Analgesic consumption also significantly decreased over time (Table 6). Compared to baseline (visit 0), a significant reduction was observed at visit 2 (*Estimate* = −0.71, *p* = 0.04) and visit 3 (*Estimate* = −2.66, *p <* 0.01), while the decrease at visit 1 did not reach statistical significance (*p* = 0.11). The THD group had slightly higher average analgesic use than the non-THD group, though the difference was not statistically significant (*p* = 0.55). The DID interaction analysis showed a significantly greater observed reduction in analgesic use in the THD group at visit 2 compared to the non-THD group (*Estimate* = −1.63, *p <* 0.01), whereas no significant difference was observed at visit 1 (*p* = 0.51) (Table 6).

**Table 6:** Estimated effects of herbal medicine on analgesic use.

	Variation	Estimate	Std. error	*t*-value	*p*-value	CI lower	CI upper
Analgesic use	Age	0.01	0.01	1.57	0.12	0.00	0.03
	Marriage	0.15	0.30	0.50	0.62	−0.44	0.74
	Smoking	0.57	0.56	1.01	0.32	−0.55	1.68
	Full-term birth	−0.33	0.32	−1.01	0.31	−0.96	0.31
	Preterm birth	0.40	0.23	1.72	0.09	−0.06	0.86
	Primary/Secondary	0.19	0.37	0.50	0.62	−0.55	0.92
	NRS at baseline	0.01	0.06	0.16	0.88	−0.10	0.12
	Analgesic use at baseline	0.57	0.06	9.80	<0.01*	0.46	0.69
	Acupuncture Tx.	−0.09	0.22	−0.44	0.66	−0.52	0.33
	Visit						
	Visit 1	−1.26	0.79	−1.59	0.11	−2.83	0.31
	Visit 2	−0.71	0.33	−2.12	0.04*	−1.37	−0.05
	Visit 3	−2.66	0.59	−4.52	<0.01*	−3.83	−1.50
	Group (ref. =non-THD)						
	THD group	0.22	0.36	0.6	0.55	−0.501	0.940
	Interaction effect						
	Herbal: Visit 1	−0.55	0.84	−0.66	0.51	−2.21	1.10
	Herbal: Visit 2	−1.63	0.47	−3.49	<0.01*	−2.55	−0.71
	(Intercept)	−0.95	1.53	−0.62	0.54*	−3.98	2.08
Linear regression Number of obs = 336 *F*(15, 131) = 29.42 Prob > F = 0.0000 R-squared = 0.6554 Root MSE = 1.4666 (Std. Err. adjusted for 132 clusters in a_newID)							

*p < 0.05.

### 3.4 Adverse events

Among the 119 participants in the THD group, 9 individuals (7.6%) reported adverse events, whereas no adverse events were observed in the control group (*n =* 16). The reported symptoms in the herbal group included indigestion, diarrhea, nausea, headache, gastrointestinal discomfort, and reproductive system-related symptoms such as increased vaginal discharge, lower abdominal pain, breast tenderness, and shortened menstrual cycles. The most frequently reported symptoms were indigestion (*n =* 3) and diarrhea (*n =* 3), followed by nausea (*n =* 2) and headache (*n =* 2). All adverse events were mild, did not interfere with daily activities, and fully resolved without any intervention. According to the CTCAE, all were classified as Grade 1 (Table 7).

**Table 7:** Adverse events reported by participants in the herbal medicine group.

Participants	Symptoms	Grade
Case 1	Indigestion, diarrhea	1
Case 2	Indigestion	1
Case 3	Indigestion	1
Case 4	Diarrhea, nausea, headache, gastrointestinal pain	1
Case 5	Nausea, headache	1
Case 6	Diarrhea	1
Case 7	Increased vaginal discharge, lower abdominal pain	1
Case 8	Shortened menstrual cycle	1
Case 9	Breast tenderness	1

## 4 Discussion

### 4.1 Key findings and implications

This multicenter, prospective descriptive registry study, designed primarily to generate hypotheses regarding outcomes potentially associated with THD use, examined individualized THD prescriptions for dysmenorrhea in real-world clinical settings. Both groups showed significant within-group reductions in menstrual pain intensity across all follow-up visits (Visit 1: Estimate = −1.35, *p <* 0.01; Visit 2: Estimate = −1.65, *p <* 0.01; Visit 3: Estimate = −3.08, *p <* 0.01). Regarding between-group differences, the DID interaction analysis showed that the THD group showed a significantly greater observed reduction in pain intensity at visit 2 compared to the non-THD group (Estimate = −1.41, *p* = 0.03), as well as a significantly greater reduction in analgesic consumption at visit 2 (Estimate = −1.63, *p <* 0.01). No statistically significant between-group difference was observed for pain duration at any visit. Critically, all between-group comparisons in this study are strictly exploratory and should not be interpreted as confirmatory evidence. The extreme imbalance between groups (THD: *n =* 119 vs. non-THD: *n =* 16) substantially reduces statistical power in the control group, inflates the variance of between-group estimates, and undermines the reliability of direct comparative inference. These preliminary between-group signals are therefore best understood as hypothesis-generating observations requiring replication in adequately powered studies. Furthermore, significant interaction effects were identified at a single time point only, with no consistent effects across other visits; this temporal instability further limits the robustness and interpretability of the between-group findings, and the possibility of random variation cannot be excluded. In particular, when statistical significance is observed at only one of multiple time points without a consistent directional pattern, such isolated findings should be interpreted with caution, as they may reflect chance variation rather than a true treatment effect. The observational design additionally precludes causal interpretation. All adverse events (7.6%) reported in the THD group were mild and resolved spontaneously without the need for additional treatment. No major safety signals were identified in this study; however, the interpretation of safety remains substantially limited, and meaningful comparative safety assessment was not feasible given the substantially underpowered control group (*n =* 16).

The significant observed reduction in analgesic consumption in the THD group (Estimate = −1.63, *p* = 0.001 at Visit 2) represents a clinically meaningful finding. NSAIDs, although widely used as first-line therapy, are associated with well-documented risks, including gastrointestinal ulceration, cardiovascular events such as myocardial infarction, and cerebrovascular events including stroke—particularly with chronic or high-dose exposure (24, 25). A large-scale meta-analysis has demonstrated a dose-dependent increase in myocardial infarction risk associated with NSAID use (24). Furthermore, a nationwide case-crossover study reported that NSAID exposure was significantly associated with elevated risks of both ischemic and hemorrhagic stroke (25). If these preliminary findings are corroborated in future studies, a reduction in reliance on NSAIDs associated with individualized THD use may be linked to a potentially more favorable tolerability profile, with possible implications for minimizing adverse event–related complications, reducing indirect economic burden, and improving long-term adherence in dysmenorrhea management.

Hyeonburikyung-tang was the most frequently prescribed THD in this study (Table 3; 43.7%, *n =* 52). This formula comprises multiple medicinal herbs, including *Angelicae Gigantis Radix* and *Paeoniae Radix Alba*, which contain bioactive constituents with anti-inflammatory and analgesic properties. It has been suggested that the combined use of these herbs may exert complementary or synergistic effects in the management of dysmenorrhea. Decursin, a major coumarin compound isolated from *Angelicae Gigantis Radix*, has been reported to attenuate inflammatory responses by inhibiting activation of nuclear factor-κB (NF-κB) signaling pathways, thereby reducing the production of pro-inflammatory mediators associated with pain (26). Paeoniflorin, the principal monoterpene glycoside of *Paeoniae Radix Alba*, has demonstrated analgesic effects through modulation of N-methyl-D-aspartate (NMDA) receptor–related pathways and has been shown to downregulate cyclooxygenase-2 (COX-2) expression, contributing to its anti-inflammatory and antinociceptive activities (27). It should be noted, however, that the mechanistic discussion above is speculative and cannot be directly linked to the clinical outcomes observed in this study, given that participants received multimodal treatments including acupuncture and conventional medicine concurrently with THD.

Recent systematic reviews have reported that herbal remedies such as cinnamon, fennel, and ginger were associated with significant observed reductions in the intensity of menstrual pain compared to controls (28–30). In particular, cinnamon has been reported to shorten the duration of pain episodes (28). In East Asian countries, including Japan, China, and Korea, multi-herbal formulations are routinely used as a primary or adjunctive treatment for dysmenorrhea. Clinical studies have reported findings suggestive of potential benefits in terms of pain-related outcomes and safety (31). These herbal medicines are believed to alleviate dysmenorrhea through mechanisms such as inhibition of prostaglandin synthesis, anti-inflammatory effects, and antispasmodic actions (31).

### 4.2 Limitation and strength

The present findings are consistent with previous clinical and systematic review studies reporting associations between herbal medicine use and reductions in pain intensity and analgesic consumption in dysmenorrhea. Notably, this study extends those observations to a real-world registry setting, providing preliminary exploratory data suggestive of a potential association between individualized THD use and reduced menstrual pain intensity and lower analgesic consumption, which may inform future hypothesis-driven research on THD as a complementary option in dysmenorrhea management.

However, this study has several limitations. First, the marked imbalance in group sizes—119 participants in the THD group versus 16 in the non-THD group—reflects the strong real-world preference for herbal medicine treatment among patients seeking care at TKM clinics in Korea, where THDs have been reimbursed under the National Health Insurance pilot project since 2020. This distributional pattern is an inherent characteristic of observational registry data collected in routine clinical practice and was not within the researchers’ control, as group allocation was determined solely by patients’ own treatment choices rather than investigator assignment. Such group imbalance is a recognized feature of real-world evidence studies and represents a trade-off between ecological validity and methodological balance. Importantly, this imbalance has substantive implications for statistical inference: it reduces statistical power in the control group, increases the variance of between-group effect estimates, and undermines the reliability of comparative analyses. Accordingly, all between-group comparisons in this study must be regarded as strictly exploratory and hypothesis-generating, not confirmatory. Although this imbalance may reduce the reliability of IPTW, increase estimate variance, and limit direct comparability between groups, we applied IPTW based on propensity scores combined with a DID framework to mitigate these effects as rigorously as possible. Sensitivity analyses with weight trimming at the 99th percentile yielded consistent results, further supporting the robustness of our estimates. Nonetheless, the relatively small overall sample size limits the statistical power and generalizability of the findings, and future studies with larger and more balanced samples are needed to validate these results.

Second, while mild adverse events were reported only in the THD group, no adverse events were observed in the non-THD group, making it difficult to compare safety profiles between groups. This limitation is also likely related to the small sample size of the control group. To overcome these limitations, future studies should employ adequately powered multi-center RCTs with sufficient allocation to both intervention and control groups. Regarding alternative designs, inclusion of a concurrent active comparator group (e.g., patients receiving conventional pharmacotherapy alone) could provide a more balanced and adequately powered comparison, representing an important direction for future research. Additionally, a single-arm pre–post design focusing exclusively on the THD group may help circumvent the power limitations associated with a small control group, although this approach would limit comparative inference. These design considerations should be taken into account in future prospective studies evaluating THDs for dysmenorrhea.

Third, as this was a real-world registry study conducted in routine clinical practice rather than a controlled RCT, it was not possible to fully control for potential confounders between the THD and non-THD groups through randomization or exclusion of concurrent therapies. In particular, confounding by indication is an inherent concern in this study: patients with more severe dysmenorrhea were more likely to seek and select THD treatment, as reflected in the significantly higher baseline NRS scores and longer pain duration observed in the THD group prior to IPTW adjustment. Although IPTW based on propensity scores was employed to balance observed covariates across groups, residual confounding from unmeasured variables such as patients’ health-seeking behavior, symptom chronicity, and personal treatment preferences cannot be fully excluded. Specifically, other Korean medicine treatments (e.g., acupuncture, moxibustion, cupping) and conventional medicine were permitted alongside THDs, reflecting actual clinical practice but introducing potential confounding from multimodal therapy effects. Furthermore, future registry studies or RCTs with larger sample sizes should consider stratified or subgroup analyses comparing THD monotherapy versus combined TKM therapy (e.g., THD plus acupuncture), which would allow for a more rigorous evaluation of the independent and additive contributions of each treatment modality. Such designs would require more granular and systematic documentation of all concurrent co-interventions in the eCRF. Although statistical methods such as IPTW were used to adjust for confounding, the influence of unmeasured variables cannot be completely ruled out. Additionally, the DID design relies on the parallel trends assumption—that the two groups would have followed similar outcome trajectories in the absence of treatment. As only a single pre-treatment measurement (Visit 0) was available in this registry, a formal statistical test of this assumption was not possible. We partially addressed this limitation by adjusting for baseline covariates likely to drive differential trends (including baseline NRS, analgesic use, and dysmenorrhea classification) and by enrolling both groups concurrently from the same clinical settings. Nevertheless, the unverified parallel trends assumption remains a limitation that should be considered when interpreting the observed associations reported in this study.

Lastly, due to the individualized nature of THD prescriptions in this study, it was not possible to characterize the clinical profile and safety of specific herbal formulas. The use of 26 different prescriptions with Gagam/Gami modifications introduces heterogeneity that may attenuate group-level observed association estimates and limit mechanistic interpretability, as different formulas target distinct TKM-defined pathophysiological patterns. Nonetheless, all prescriptions were selected within the Korean Medicine CPG for Dysmenorrhea framework and prepared under GMP standards, providing a meaningful level of standardization within routine TKM practice. Future studies should employ standardized formulations or formula-stratified designs for formula-specific and mechanistic evaluation. To inform future recommendations regarding THD in dysmenorrhea management, future studies should aim to standardize herbal formulations for more rigorous and consistent evaluation of potential associations.

### 4.3 Future suggestion

Real-world clinical registry data offer greater detail and scope than health insurance claims data and enable long-term follow-up. Future studies with larger samples and broader TKM networks would improve generalizability, and longitudinal registry-based follow-up studies are needed to examine potential long-term associations between THD use and outcomes in dysmenorrhea. Patients with dysmenorrhea often choose between conventional medicine, TKM, or a combination of both. Registry-based comparative effectiveness research evaluating TKM monotherapy versus integrative care is therefore warranted, and future studies should incorporate economic evaluations given the additional time and cost burdens of integrative approaches.

Large-scale data collection from real-world clinical settings, combined with artificial intelligence (AI) and big data analytics, may enable personalized herbal prescriptions based on individual characteristics such as constitution, symptoms, and medical history (32). Furthermore, AI models may contribute to standardizing prescription selection, herbal composition, and dosage, thereby reducing clinical variability and enhancing treatment consistency. The use of complex herbal combinations tailored to individual patients is a characteristic feature of traditional medicine practices in East Asian countries, including Korea, China, and Japan (33). Therefore, international collaboration across East Asia is essential to develop a standardized multicenter RCT protocol, which would strengthen the clinical evidence base for THD in dysmenorrhea.

## 5 Conclusion

This multicenter prospective descriptive registry study, conceived as a hypothesis-generating investigation, provides preliminary exploratory evidence that individualized THD prescriptions may be associated with greater observed reductions in menstrual pain intensity and analgesic use in women with dysmenorrhea in real-world clinical settings. All adverse events reported were mild and resolved spontaneously without additional treatment. No major safety signals were identified; however, the interpretation of safety in this study remains substantially limited, and comparative safety conclusions cannot be drawn given the extremely small and underpowered control group (*n =* 16). Several additional limitations should be considered when interpreting these findings, including the substantial group imbalance inherent to real-world observational data (reflecting patient treatment preference in routine TKM practice), the relatively small sample size, non-randomized study design, and heterogeneity of individualized herbal prescriptions. Larger-scale, adequately powered trials with standardized formulations and rigorous comparative designs are warranted to confirm these preliminary findings. Overall, this study provides a suggestive signal that traditional herbal medicine use may be linked to more favorable pain-related outcomes in women with dysmenorrhea in real-world clinical settings, with mild adverse events resolving spontaneously in the THD group. Larger-scale, adequately powered multicenter trials, clinical studies applying standardized herbal formulations, precision medicine approaches, and international collaborative efforts are needed to replicate and evaluate these preliminary exploratory associations.

## Data Availability

The original contributions presented in the study are included in the article/Supplementary material, further inquiries can be directed to the corresponding author.

## Footnotes

1
http://ecrf.nikom.or.kr
