# Correction: Herbal medicine as a complementary therapy for dysmenorrhea: effects on pain and reduction of analgesic use

**DOI:** 10.3389/fmed.2026.1967756

**Published:** 2026-09-04

**Authors:** Soo-Hyun Sung, Kyeore Bae, Hyein Jeong, Dong-Il Kim, Minjung Park

**Affiliations:** 1 School of Korean Medicine, Wonkwang University, Iksan, Republic of Korea; 2 National Agency for Korean Medicine Innovative Technologies Development, National Institute for Korean Medicine Development, Seoul, Republic of Korea; 3 Department of Preventive Medicine, College of Korean Medicine, Kyung Hee University, Seoul, Republic of Korea; 4 Department of Obstetrics and Gynecology, Dongguk University Ilsan Korean Medicine Hospital, Goyang, Republic of Korea; 5 Department of Preventive Medicine, College of Korean Medicine, Gachon University, Seongnam, Republic of Korea

**Keywords:** dysmenorrhea, herbal decoction, herbal medicine, real-world evidence (RWE), registry study

While reviewing the published tables, it was found that the nine adjustment-variable rows (Age, Marital status, Smoking, Full-term birth, Preterm birth, Primary/Secondary dysmenorrhea classification, outcomes at baseline) had been transcribed from the wrong regression output when the tables were assembled, so the values shown did not correspond to each table's own model. The three tables have been corrected so that every row — adjustment variables and key exposure variables alike — comes from the single, correct regression underlying that table.

There was a mistake in Table 4 as published. The corrected Table 4 appears below.

**Table 4:** Estimated effects of herbal medicine on severity of menstrual pain (NRS). *p < 0.05.

Linear regression			Number of obs = 338				
			F(15, 131) = 23.10				
			Prob > F = 0.0000				
			R-squared = 0.5971				
			Root MSE = 1.6215				
			(Std. Err. adjusted for 132 clusters in a_newID)				
	Variation	Estimate	Std. Error	*t*-value	*p*-value	CI lower	CI Upper
Severity of Menstrual Pain (NRS)	Age	0.00	0.01	0.25	0.80	−0.02	0.03
	Marriage	−0.04	0.47	−0.08	0.94	−0.96	0.89
	Smoking	−0.65	0.68	−0.95	0.34	−1.99	0.70
	Full-term birth	−0.53	0.49	−1.08	0.28	−1.50	0.44
	Preterm birth	0.82	0.25	3.30	<0.01*	0.33	1.31
	Primary/Secondary	0.37	0.46	0.81	0.42	−0.54	1.29
	NRS at baseline	0.66	0.08	8.65	<0.01*	0.51	0.81
	Analgesic use at baseline	0.02	0.03	0.49	0.63	−0.05	0.08
	Acupuncture Tx.	−0.27	0.26	−1.02	0.31	−0.78	0.25
	Visit						
	Visit 1	−1.35	0.36	−3.77	<0.01*	−2.06	−0.64
	Visit 2	−1.65	0.52	−3.15	<0.01*	−2.69	−0.61
	Visit 3	−3.08	0.80	−3.87	<0.01*	−4.66	−1.51
	Group (ref. = non-THD)						
	THD group	−0.17	0.24	−0.72	0.47	−0.63	0.30
	Interaction effect						
	Herbal: Visit 1	−0.75	0.43	−1.75	0.08	−1.59	0.10
	Herbal: Visit 2	−1.41	0.64	−2.21	0.03*	−2.68	−0.15
	(Intercept)	3.18	2.01	1.58	0.12	−0.80	7.16

There was a mistake in Table 5 as published. The corrected Table 5 appears below.

**Table 5:** Estimated effects of herbal medicine on duration of menstrual pain. *p < 0.05.

Linear regression			Number of obs = 355				
			F(15, 131) = 11.35				
			Prob > F = 0.0000				
			R-squared = 0.3148				
			Root MSE = 17.948				
			(Std. Err. adjusted for 132 clusters in a_newID)				
	Variation	Estimate	Std. Error	*t*-value	*p*-value	CI lower	CI Upper
Duration of Menstrual Pain	Age	−0.13	0.13	−0.95	0.34	−0.39	0.14
	Marriage	2.17	3.81	0.57	0.57	−5.37	9.71
	Smoking	7.58	3.76	2.02	0.05*	0.14	15.01
	Full-term birth	−0.30	4.30	−0.07	0.94	−8.82	8.21
	Preterm birth	3.65	1.63	2.24	0.03*	0.42	6.87
	Primary/Secondary	−0.18	3.94	−0.05	0.96	−7.97	7.60
	NRS at baseline	−0.25	0.62	−0.40	0.69	−1.47	0.98
	Analgesic use at baseline	2.52	0.58	4.38	<0.01*	1.38	3.66
	Acupuncture Tx.	4.90	3.51	1.39	0.17	−2.05	11.85
	Visit						
	Visit 1	−13.808	5.922	−2.33	0.02*	−25.524	−2.092
	Visit 2	−13.679	4.156	−3.29	<0.01*	−21.901	−5.458
	Visit 3	−14.622	5.065	−2.89	0.01*	−24.642	−4.603
	Group (ref. = non-THD)						
	THD group	9.403	4.690	2.01	0.05*	0.126	18.681
	Interaction effect						
	Herbal: Visit 1	−2.363	6.390	−0.37	0.71	−15.005	10.279
	Herbal: Visit 2	−1.378	5.439	−0.25	0.80	−12.139	9.382
	(Intercept)	−3.942	12.909	−0.31	0.76	−29.479	21.596

There was a mistake in Table 6 as published. The corrected Table 6 appears below.

**Table 6:** Estimated effects of herbal medicine on analgesic use. *p < 0.05.

Linear regression			Number of obs = 336				
			F(15, 131) = 29.42				
			Prob > F = 0.0000				
			R-squared = 0.6554				
			Root MSE = 1.4666				
			(Std. Err. adjusted for 132 clusters in a_newID)				
	Variation	Estimate	Std. Error	*t*-value	*p*-value	CI lower	CI Upper
Analgesic Use	Age	0.01	0.01	1.57	0.12	0.00	0.03
	Marriage	0.15	0.30	0.50	0.62	−0.44	0.74
	Smoking	0.57	0.56	1.01	0.32	−0.55	1.68
	Full-term birth	−0.33	0.32	−1.01	0.31	−0.96	0.31
	Preterm birth	0.40	0.23	1.72	0.09	−0.06	0.86
	Primary/Secondary	0.19	0.37	0.50	0.62	−0.55	0.92
	NRS at baseline	0.01	0.06	0.16	0.88	−0.10	0.12
	Analgesic use at baseline	0.57	0.06	9.80	<0.01*	0.46	0.69
	Acupuncture Tx.	−0.09	0.22	−0.44	0.66	−0.52	0.33
	Visit						
	Visit 1	−1.26	0.79	−1.59	0.11	−2.83	0.31
	Visit 2	−0.71	0.33	−2.12	0.04*	−1.37	−0.05
	Visit 3	−2.66	0.59	−4.52	<0.01*	−3.83	−1.50
	Group (ref. = non-THD)						
	THD group	0.22	0.36	0.6	0.55	−0.501	0.940
	Interaction effect						
	Herbal: Visit 1	−0.55	0.84	−0.66	0.51	−2.21	1.10
	Herbal: Visit 2	−1.63	0.47	−3.49	<0.01*	−2.55	−0.71
	(Intercept)	−0.95	1.53	−0.62	0.54	−3.98	2.08

A correction has been made to the **3 Results**, *3.3 Effectiveness outcomes, 3.3.1. Severity of Menstrual Pain:*

“A total of 338 observations from 132 unique participants were analyzed. Standard errors were adjusted for 132 clusters to provide valid inference despite the intra-cluster correlation inherent in repeated measures. Pain severity decreased significantly over time. Compared to baseline (visit 0), significant reductions were observed at visit 1 (*Estimate* = −13.81, *p* = 0.02), visit 2 (*Estimate* = −13.68, *p* *<* 0.01), and visit 3 (*Estimate* = −14.62, *p* = 0.01). Between-group comparison revealed that the herbal medicine group had a significantly longer duration of menstrual pain than the control group (*Estimate* = 9.40, *p* *<* 0.05). However, no statistically significant interaction effects were observed at visit 1 or visit 2 (*p* > 0.7), indicating no significant between-group difference in pain duration (**Table 5**).

The corrected paragraph is:

“A total of 338 observations from 132 unique participants were analyzed. Standard errors were adjusted for 132 clusters to provide valid inference despite the intra-cluster correlation inherent in repeated measures. Menstrual pain severity (NRS) decreased significantly over time. Compared to baseline (visit 0), significant reductions were observed at visit 1 (*Estimate* = −1.35, *p* < 0.01), visit 2 (*Estimate* = −1.65, *p* < 0.01), and visit 3 (*Estimate* = −3.08, *p* < 0.01). The main effect of group was not statistically significant, indicating no overall difference in baseline pain severity between the THD and control groups (*Estimate* = −0.17, *p* = 0.47). The DID interaction analysis showed a significantly greater reduction in pain severity in the THD group at visit 2 compared to the control group (*Estimate* = −1.41, *p* = 0.03), whereas the difference at visit 1 was not statistically significant (*Estimate* = −0.75, *p* = 0.08) (Table 4).”

There was an error in the table citations in section 3.3. Only Table 4 was cited when Tables 4–6 should have been cited. A correction has been made to the **3 Results**, *3.3. Effectiveness outcomes*, paragraph 1:

The sentence previously read: “**Table 4** presents the estimated effects of THD on three menstrual pain–related outcomes (pain severity, pain duration, and analgesic consumption) over time.”

The corrected sentence is: “**Tables 4 to 6** present the estimated effects of THD on three menstrual pain–related outcomes (pain severity, pain duration, and analgesic consumption) over time.”

There was an error in the table citation in section 3.3.3. A correction has been made to **3 Results**, *3.3 Effectiveness outcomes, 3.3.3. Analgesic Consumption*:

The sentence previously read: “Analgesic consumption also significantly decreased over time (**Table 4**).”

The corrected sentence is: “Analgesic consumption also significantly decreased over time (Table 6).”

An incorrect Funding statement was provided. The correct funder is the Korean Medicine Innovative Technology Development Project. The correct funding statement reads:

“The author(s) declared that financial support was received for this work and/or its publication. This work was supported by the Korean Medicine Innovative Technology Development Project through the Korea Health Industry Development Institute (KHIDI), funded by the Ministry of Health & Welfare, Republic of Korea (grant number: RS-2026-25531949).”

The original version of this article has been updated.

