# Supplementary material for: Herbal medicine as a complementary therapy for dysmenorrhea: effects on pain and reduction of analgesic use
Source: Front Med (Lausanne). 2026 May 20;13:1719823. doi: 10.3389/fmed.2026.1719823 (PMC13230185; doi:10.3389/fmed.2026.1719823)
Supplement: Supplementary file 1 [file Table_1.docx]

Supplementary 1 Sensitivity analysis of NRS

| Linear regression Number of obs = 338  F(15, 131) = 22.77  Prob > F = 0.0000  R-squared = 0.5866  Root MSE = 1.6328 (Std. Err. adjusted for 132 clusters in a_newID) | | | | | | | |
| --- | --- | --- | --- | --- | --- | --- | --- |
|  | **Variation** | **Estimate** | **Std. Error** | **t value** | **p-value** | **CI lower** | **CI Upper** |
| Severity of  Menstrual Pain (NRS) | Age | 0.00 | 0.01 | 0.34 | 0.74 | -0.02 | 0.03 |
|  | Marriage | -0.04 | 0.47 | -0.10 | 0.92 | -0.97 | 0.88 |
|  | Smoking | -0.63 | 0.68 | -0.94 | 0.35 | -1.97 | 0.71 |
|  | Full-term birth | -0.56 | 0.49 | -1.15 | 0.25 | -1.52 | 0.40 |
|  | Preterm birth | 0.81 | 0.25 | 3.24 | <0.01* | 0.31 | 1.30 |
|  | Primary/Secondary | 0.37 | 0.46 | 0.80 | 0.42 | -0.54 | 1.28 |
|  | NRS at baseline | 0.65 | 0.08 | 8.55 | <0.01* | 0.50 | 0.81 |
|  | Analgesic use at baseline | 0.02 | 0.03 | 0.59 | 0.55 | -0.05 | 0.09 |
|  | Acupuncture Tx. | -0.30 | 0.26 | -1.16 | 0.25 | -0.81 | 0.21 |
|  | Visit |  |  |  |  |  |  |
|  | Visit 1 | -1.30 | 0.36 | -3.67 | <0.01* | -2.01 | -0.60 |
|  | Visit 2 | -1.61 | 0.52 | -3.10 | <0.01* | -2.63 | -0.58 |
|  | Visit 3 | -3.08 | 0.80 | -3.88 | <0.01* | -4.66 | -1.51 |
|  | Group (ref. =non-THD) |  |  |  |  |  |  |
|  | THD group | -0.12 | 0.23 | -0.50 | 0.62 | -0.57 | 0.34 |
|  | Interaction effect |  |  |  |  |  |  |
|  | Herbal:Visit 1 | -0.79 | 0.42 | -1.88 | 0.06 | -1.63 | 0.04 |
|  | Herbal:Visit 2 | -1.46 | 0.63 | -2.30 | 0.02* | -2.71 | -0.20 |
|  | (Intercept) | 2.01 | 1.56 | 0.12 | -0.83 | 7.11 | 2.01 |

Supplementary 2 Sensitivity analysis of Menstrual Pain Duration

| Linear regression  Number of obs = 355  F(15, 131) = 11.17  Prob > F = 0.0000  R-squared = 0.3112  Root MSE = 18.233  (Std. Err. adjusted for 132 clusters in a_newID) | | | | | | | |
| --- | --- | --- | --- | --- | --- | --- | --- |
|  | **Variation** | **Estimate** | **Std. Error** | **t value** | **p-value** | **CI lower** | **CI Upper** |
| Duration of Menstrual Pain | Age | -0.12 | 0.13 | -0.94 | 0.35 | -0.38 | 0.14 |
|  | Marriage | 2.16 | 3.82 | 0.56 | 0.57 | -5.40 | 9.72 |
|  | Smoking | 7.61 | 3.76 | 2.02 | 0.05 | 0.17 | 15.05 |
|  | Full-term birth | -0.37 | 4.32 | -0.09 | 0.93 | -8.93 | 8.18 |
|  | Preterm birth | 3.63 | 1.62 | 2.24 | 0.03* | 0.43 | 6.84 |
|  | Primary/Secondary | -0.19 | 3.93 | -0.05 | 0.96 | -7.97 | 7.59 |
|  | NRS at baseline | -0.26 | 0.62 | -0.42 | 0.67 | -1.50 | 0.97 |
|  | Analgesic use at baseline | 2.53 | 0.58 | 4.35 | <0.01* | 1.38 | 3.68 |
|  | Acupuncture Tx. | 4.82 | 3.58 | 1.35 | 0.18 | -2.26 | 11.89 |
|  | Visit |  |  |  |  |  |  |
|  | Visit 1 | -13.46 | 6.41 | -2.10 | 0.04* | -26.14 | -0.78 |
|  | Visit 2 | -13.46 | 4.39 | -3.07 | <0.01* | -22.13 | -4.78 |
|  | Visit 3 | -14.62 | 5.07 | -2.89 | 0.01* | -24.64 | -4.60 |
|  | Group (ref. =non-THD) |  |  |  |  |  |  |
|  | THD group | 9.65 | 4.95 | 1.95 | 0.05* | -0.14 | 19.43 |
|  | Interaction effect |  |  |  |  |  |  |
|  | Herbal:Visit 1 | -2.71 | 6.84 | -0.40 | 0.69 | -16.25 | 10.83 |
|  | Herbal:Visit 2 | -1.60 | 5.60 | -0.29 | 0.78 | -12.68 | 9.49 |
|  | (Intercept) | -4.18 | 12.91 | -0.32 | 0.75 | -29.71 | 21.36 |

Supplementary 3 Sensitivity analysis of Analgesic Use

| Linear regression  Number of obs = 336  F(15, 131) = 28.79  Prob > F = 0.0000  R-squared = 0.6554  Root MSE = 1.4798  (Std. Err. adjusted for 132 clusters in a_newID) | | | | | | | |
| --- | --- | --- | --- | --- | --- | --- | --- |
|  | **Variation** | **Estimate** | **Std. Error** | **t value** | **p-value** | **CI lower** | **CI Upper** |
| Duration of Menstrual Pain | Age | 0.01 | 0.01 | 1.58 | 0.12 | 0.00 | 0.03 |
|  | Marriage | 0.15 | 0.30 | 0.50 | 0.62 | -0.44 | 0.75 |
|  | Smoking | 0.57 | 0.56 | 1.00 | 0.32 | -0.55 | 1.68 |
|  | Full-term birth | -0.32 | 0.33 | -0.99 | 0.32 | -0.97 | 0.32 |
|  | Preterm birth | 0.40 | 0.23 | 1.73 | 0.09 | -0.06 | 0.86 |
|  | Primary/Secondary | 0.19 | 0.37 | 0.50 | 0.62 | -0.55 | 0.92 |
|  | NRS at baseline | 0.01 | 0.06 | 0.17 | 0.87 | -0.10 | 0.12 |
|  | Analgesic use at baseline | 0.57 | 0.06 | 9.78 | <0.01* | 0.46 | 0.69 |
|  | Acupuncture Tx. | -0.09 | 0.22 | -0.42 | 0.68 | -0.52 | 0.34 |
|  | Visit |  |  |  |  |  |  |
|  | Visit 1 | -1.26 | 0.81 | -1.56 | 0.12 | -2.86 | 0.33 |
|  | Visit 2 | -0.71 | 0.35 | -2.05 | 0.04* | -1.40 | -0.02 |
|  | Visit 3 | -2.66 | 0.59 | -4.52 | <0.01* | -3.83 | -1.50 |
|  | Group (ref. =non-THD) |  |  |  |  |  |  |
|  | THD group | 0.21 | 0.38 | 0.56 | 0.57 | -0.54 | 0.97 |
|  | Interaction effect |  |  |  |  |  |  |
|  | Herbal:Visit 1 | -0.55 | 0.85 | -0.64 | 0.52 | -2.23 | 1.14 |
|  | Herbal:Visit 2 | -1.62 | 0.48 | -3.41 | <0.01* | -2.57 | -0.68 |
|  | (Intercept) | -0.95 | 1.53 | -0.62 | 0.54 | -3.98 | 2.08 |
